# A core-based XRF scanning workflow for continuous measurement of mineralogical variations in clastic reservoirs

**DOI:** 10.1016/j.mex.2022.101928

**Published:** 2022-11-15

**Authors:** Arif Hussain, Khalid Al-Ramadan

**Affiliations:** Department of Geosciences, College of Petroleum Engineering and Geosciences (CPG), King Fahd University of Petroleum and Minerals, 31261, Dhahran, Saudi Arabia

**Keywords:** Itrax core scanning, Core plug, Log-ratio calibration, Elemental profile, Clastic reservoir

## Abstract

Clastic reservoir core is routinely characterized using conventional, destructive spot sampling techniques. Whilst spot sampling provides useful textural and compositional information, the samples are typically widely spaced (∼ 25 cm), and hence continuous variation in rock composition/texture is not fully captured. It is therefore important to develop higher-resolution rock characterization techniques. In this paper, compositional data from a micro x-ray fluorescence (µXRF) scanner (hereafter Itrax) was used to demonstrate near-continuous (at 200 µm resolution) mineralogical variations in an ancient sandstone core. Traditionally, Itrax was used for compositional profiling of soft sediment cores, with limited use of this technology with rock cores. The acquired XRF data reveal subtle but systematic vertical compositional/textural trends, that may reflect distribution of major sandstone forming mineral grains, clay minerals, diagenetic carbonate cements and identification of new textural subdivisions in sandstones, which would not otherwise be seen with conventional plug analysis, implying the importance of high-resolution core scanning techniques for continuous measurement of mineralogy in clastic reservoirs.

• Limited literature could be found whereby the capability of Itrax scanning has been extended to ancient, clastic cores.
• Itrax is a non-destructive technique, where elemental composition is obtained directly at the surface of a split core at resolution significantly higher than conventional plug based techniques.
• Itrax data acquisition is a quick process (few hours) and mineral distribution trends can be used as guide for further specialized sampling and detailed investigation of clastic reservoirs.

## Graphical abstract

**Figure:**
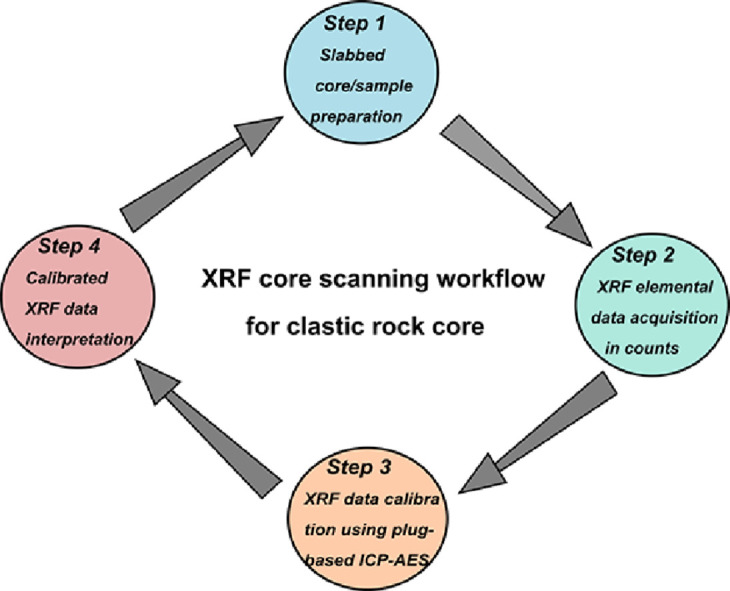

Specifications Table

**Table:** 

Subject area	Earth and Planetary Sciences
More specific subject area:	Reservoir characterization
Name of your method:	XRF core scanning
Name and reference of original method:	A. Hussain, P. Haughton, P. Shannon, J. Turner, C. Pierce, A. Obradors-Latre, S. Barker, O. Martinsen, High-resolution X-ray fluorescence profiling of hybrid event beds: implications for sediment gravity flow behaviour and deposit structure, Sedimentology 67(2020) 2850–2882. https://doi.org/10.1111/sed.12722^[^^3^^]^.
Resource availability:	XRF core scanner, slabbed rock core

## Background

Itrax is an energy dispersive, micro x-ray fluorescence (µXRF) scanning instrument that returns elemental abundances as intensities (counts per second, cps) with variable detection limits depending on the atomic weight. The core scans employ a variable step-size ranging from cms down to 100 microns [1], [2], [3], [4], [5], [6]. The key advantage of Itrax core scanning over conventional XRF analysis is that elemental composition is obtained directly at the surface of a split sediment core [6] at much higher resolution than other conventional destructive [7], [8], [9], [10], thus it provides near-continuous compositional records of element intensities. Itrax has been traditionally used to capture sub-mm scale compositional trends in shallow soft sediment cores [2]. However, there has been only limited use of this technology in the deeper subsurface and with rock cores [3,4]. The current study was therefore conceived to develop and assess a new XRF based workflow for characterizing vertical compositional trends in clastic sedimentary rock cores. Further details on Itrax instrument are provided in [1].

## High-resolution mineralogical analysis workflow

**Fig. 1:**
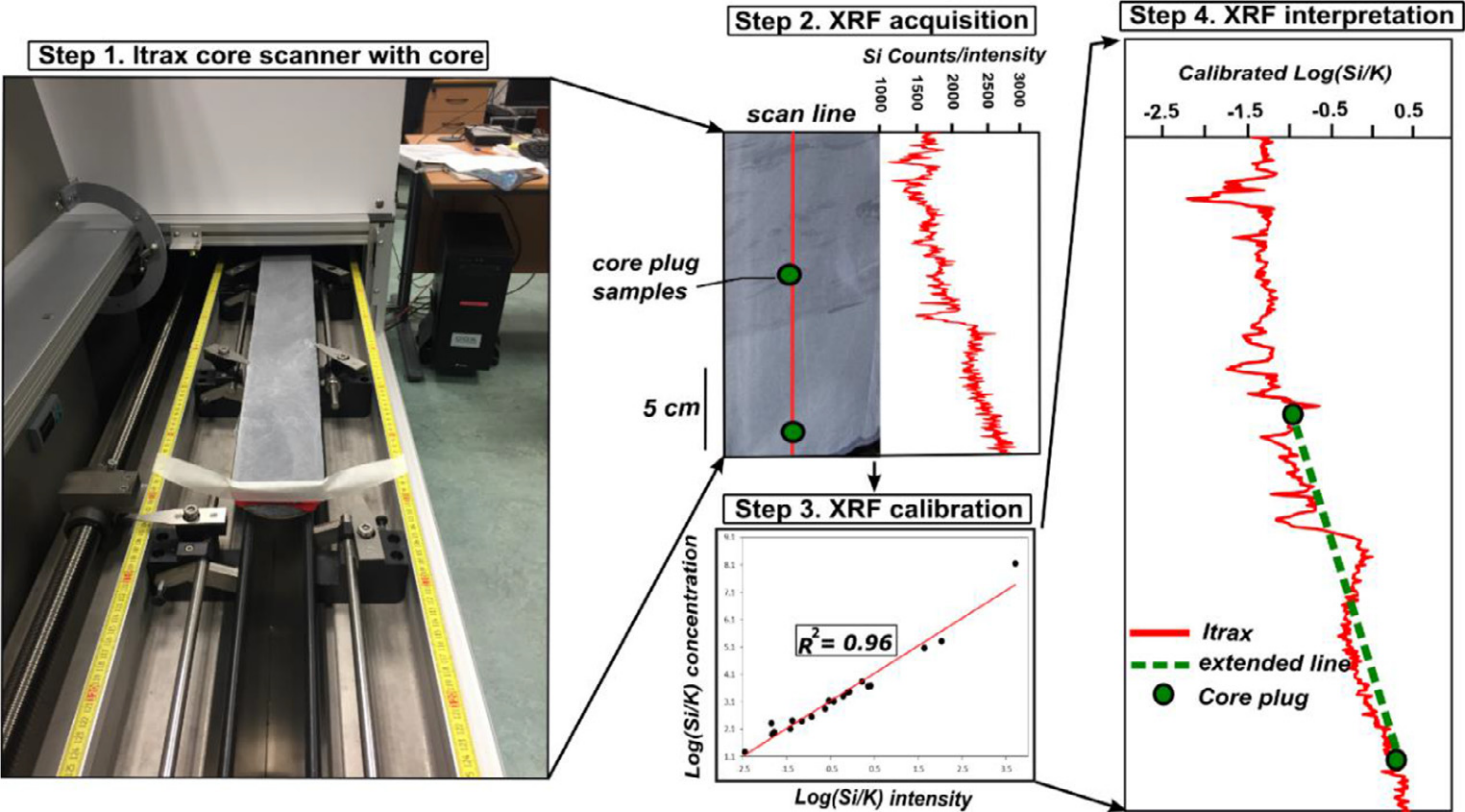
Flow chart illustrating the four main steps undertaken to perform continuous mineralogical measurements on clastic core in this study. The green dotted lines joining core plug points cannot be necessarily extrapolated.

The purpose of this study is to devise a workflow for the Itrax core scanning to document high-resolution mineralogical variations in sandstone rock core. Some of the other tools employed (petrography etc.) in this study are already well established in terms of their application to rock cores [8], [9], [10], whereas Itrax scanning workflow has had to be adapted and developed for clastic rock cores. The key steps involved in developing the Itrax workflow for clastic rocks are discussed and illustrated in Fig. 1.

*(i) Sampling*

The Itrax workflow commonly requires slabbed rock core as sample for scanning. This study uses sedimentological and supplementary, raw compositional data acquired on split drill cores from deep-marine Pennsylvanian Ross Sandstone Formation in the west of Ireland analysed by [3,4,7]. The Ross Formation was cored (funded by Equinor) recently to better understand the deposition of deep-marine sandstones. Coring has identified a wide range of deposits including cleaner (clay-poor) and clay-rich sandstones [7]. Detailed petrographical characterization of different sandstones from the Ross suggests that it is a useful prototype succession to explore high-resolution Itrax profiling because sandstones have relatively simple bimodal mineralogy of quartz sand and clay [3,4,7] and hence distribution of minerals and clues to the sandstone origin can be better constrained.

*(ii) XRF data acquisition*

The core was progressively moved past a 20 mm wide and 0.2 mm thick molybdenum (Mo) beam and an XRF detector with an 8 mm wide nozzle. The depth to which the beam penetrates varies and is likely to be a few microns for light elements, and up to 100 microns for heavy elements [1]. The instrument was operated at 30 kV and 50 mA, with a count time of 15 seconds and step size of 200 µm. The major and trace element data obtained were internally processed using fitting procedures in the Q-Spec spectral analysis software package programmed with Itrax scanner. Operation of the software involved selecting elements to be extracted from the spectra, with any spurious or unnecessary elemental choices or incorrect fitting parameters adjusted through a batch-controlled post-processing of the spectra [1]. Compositional scans were integrated with the core by using optical images acquired by the scanner and by aligning breaks between core pieces, which are obvious on the XRF counts/elemental profile as *‘backsteps’*.

*(iii) XRF calibration*

Itrax scanner outputs compositional data as raw intensities for each element and calibration to actual elemental concentrations requires quantitative geochemical analysis of representative co-located samples (collected over the 20 mm high window coinciding with the plug diameter) of the bulk rock [6]. This is an important step in substantiating the results. Element concentrations for the 19 powdered samples were measured by ICP-AES. A homogenised powder sample (0.2 g) from the core was added to lithium metaborate/lithium tetraborate flux (0.9 g), mixed well and fused in a furnace at 1000°C. The resulting melt was then cooled and dissolved in 100 mL of 4% nitric acid and 2% hydrochloric acid. This solution was then analyzed by ICP-AES and the results were corrected for spectral inter-element interferences (if any). Oxide (wt%) and trace element (ppm) concentrations were calculated from the concentrations of each element measured in the solution.

Many studies use dimensionless element ratios to monitor core compositional changes as this circumvents unit sum constraints often associated with XRF data [1,3,5]. Authors [6] introduced a method for calibration of XRF-core scanners using log-ratio calibration equations (LRCEs) based on cross-correlation of scanner log-ratio intensities and log-ratios of independently measured element concentrations (Table 1). The LRCE-based calibration can significantly reduce the effects of overlap between elements and problems due to closure inherent in compositional datasets, particularly when there is variation in an element that cannot be measured by the scanner [6]. However, the downside of this approach is that interpretation of log ratio trends is less intuitive [3].

**Table 1:** Log-ratios of Itrax intensities and ICP-AES concentrations collected from 19 core plug samples for calibration. For further details refer to [3] supplementary data Table S1.

Sample	Log(Si/K) Itrax	Log(Si/K) ICP-AES	Log(Ca/K) Itrax	Log(Ca/K) ICP-AES	Log(Fe/K) Itrax	Log(Fe/K) ICP-AES	Log(Ti/K) Itrax	Log(Ti/K) ICP-AES	Log(Zr/K) Itrax	Log(Zr/K) ICP-AES	K_2_O Itrax	K_2_O wt% ICP-AES
1	-0.6	1.3	-0.69	-1.01	1.53	0.41	-0.03	-0.56	-0.93	1.76	4583	2.84
2	0.88	2.56	1.41	1.01	2.13	1.05	0.42	0	0.07	2.91	450	0.24
3	0.71	2.45	0.83	0.58	1.93	0.83	0.32	-0.07	-0.04	2.83	679	0.32
4	-0.24	1.62	-0.62	-0.88	1.54	0.45	0.07	-0.39	-0.73	2.15	3957	1.8
5	-0.05	1.75	-0.5	-0.7	1.66	0.57	0.13	-0.32	-0.62	2.27	2881	1.39
6	-1.07	0.8	-1.04	-1.5	1.08	0.05	-0.19	-0.65	-1.22	1.66	8758	6.66
7	0.09	1.92	0.03	-0.06	1.86	0.70	0.17	-0.27	-0.46	2.38	1627	0.95
8	1.61	3.8	2.61	2.44	3.97	3.20	1.84	1.59	1.04	4.21	30	0.01
9	-0.81	1.26	-1.03	-1.01	1.31	0.29	-0.17	-0.54	-1.08	1.83	6943	3.44
10	-0.62	1.17	-0.96	-1.23	1.33	0.15	-0.12	-0.63	-1.06	1.66	7036	4.21
11	-0.78	1.12	-1.18	-1.33	1.16	0.08	-0.2	-0.66	-1.2	1.57	9161	4.71
12	-0.8	1.08	-1.13	-1.31	1.18	0.06	-0.2	-0.67	-1.2	1.62	9231	4.74
13	-0.2	1.55	-0.33	-0.52	1.62	0.47	0.06	-0.46	-0.69	2.11	3583	2.04
14	-0.27	1.49	-0.28	-0.56	1.55	0.39	0.02	-0.47	-0.8	2	4065	2.3
15	-0.51	1.29	-0.86	-1.12	1.34	0.19	-0.05	-0.56	-0.9	1.8	6159	3.46
16	-0.03	1.76	-0.24	-0.48	1.71	0.63	0.15	-0.32	-0.4	2.44	2585	1.34
17	0.18	1.86	0.1	-0.2	1.82	0.64	0.23	-0.29	-0.38	2.37	1835	1.11
18	0.15	1.85	0.09	-0.21	1.81	0.63	0.2	-0.29	-0.43	2.29	1907	1.1
19	-0.19	1.61	-0.29	-0.55	1.55	0.46	0.07	-0.41	-0.68	2.02	3649	1.82

The LRCE-based calibration undertaken for the studied core involved additive log transformation (alr) of intensity and concentration data following [6]. Regression analysis showed strong correlations between intensity and concentration for all the key elements as indicated by high R^2^ values (typically > 0.95; Table 2). Based on the goodness-of fit (highest R^2^ value with maximum number of elements), K was selected as optimum denominator for the calibrated log ratio data. Although normally log-ratios would be used to monitor vertical compositional changes, direct regression of scanner intensity and element concentrations measured on the plugs produce strong linear relationships (Table 2). A R^2^ of 0.93 for the core plug K_2_O, when regressed against corresponding core scanner intensities justifies the use of calibrated K_2_O profiles in this instance. High R^2^ values for all the measured elements suggest that studied cores do not seem to suffer from some of the calibration issues observed elsewhere in soft-sediment and water saturated cores. This likely reflects the relatively simple quartzose composition, minimal porosity (<1%), smooth cut core surfaces and the absence of interstitial pore fluids. [3] stated that the studied rocks are well-suited for elemental profiling because these rock dominantly comprise a simple bimodal mix of dominantly quartz-rich sand and K and Fe-rich clay phases. Hence, log(Si/K) is a useful measure of variations in illite plus mica relative to quartz, and K_2_O is a good proxy for illite (plus detrital muscovite) profiles in these rocks. Similarly, log(Fe/K) can be used a proxy for chlorite and log(Ca/K) is a guide to the distribution of diagenetic calcite.

**Table 2:** R^2^ values (based on cross correlation of intensities and concentration of elements presented in Table. 1) for determination of optimum log-ratio denominators. Goodness-of-fit suggest that ‘K’ is the best denominator for log-ratios. For further details refer to [3].

Element	Si	K	Ca	Fe	Ti	Zr
Si	0.96	0.96	0.87	0.95	0.96	0.58
K	0.96	0.93	0.98	0.99	0.99	0.97
Ca	0.87	0.99	0.99	0.90	0.96	0.92
Fe	0.95	0.99	0.90	0.95	0.95	0.86
Ti	0.95	0.99	0.96	0.94	0.99	0.88
Zr	0.58	0.97	0.92	0.86	0.88	0.97

*(iv) Itrax data interpretation*

**Fig. 2:**
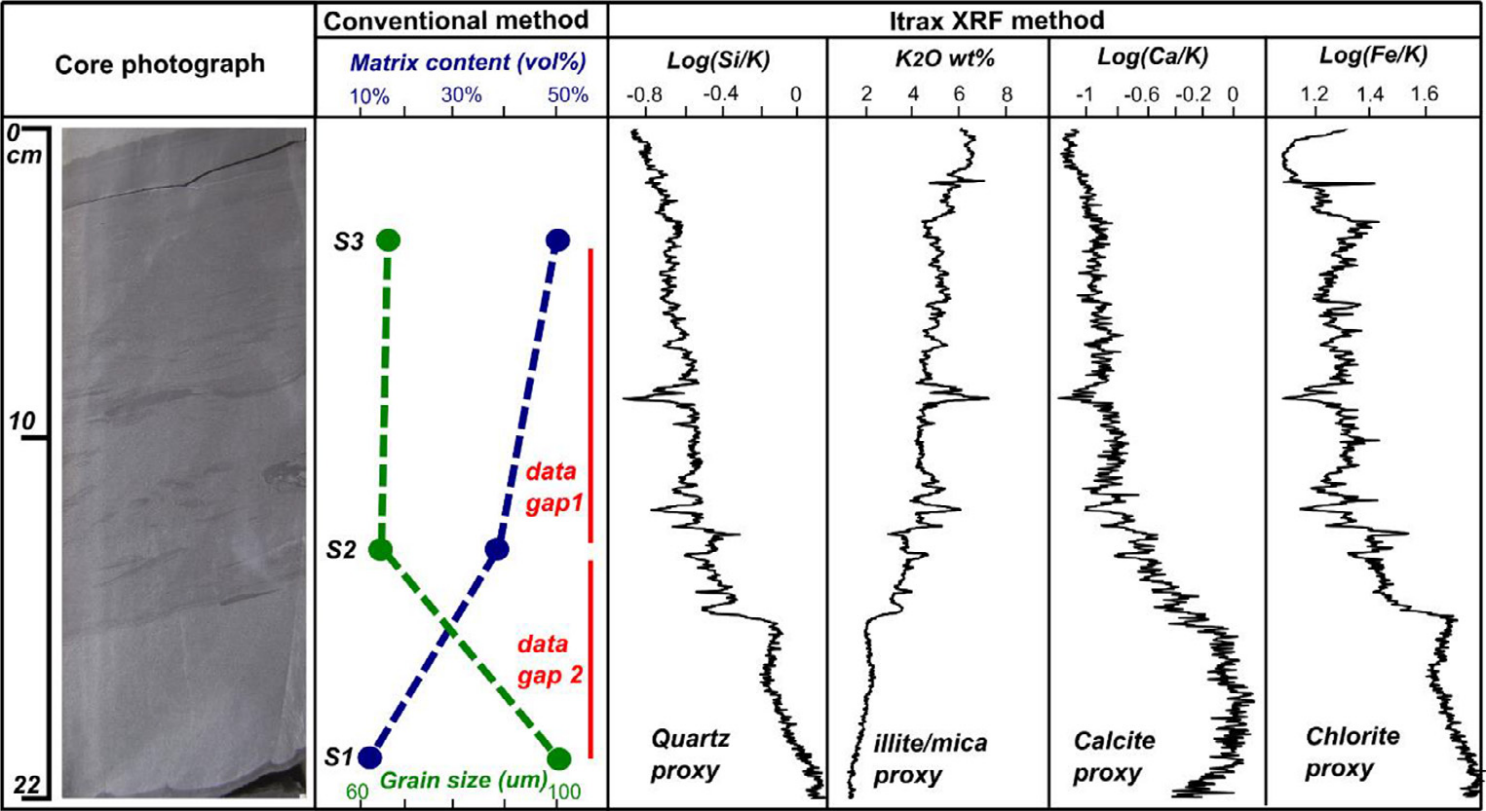
Core photograph, petrography based grain size and matrix content curves, and XRF element and element ratio-profiles for a 22 cm thick, clay-rich deep-marine sandstone bed from Ross sandstone Formation. Core photograph is obtained from [7] and composition data is obtained from [3,4]. Grain-size and matrix quantification curves from coincident thin sections (represented as S1-S3) cannot be necessarily extrapolated, leaving behind data gaps (represented as data gaps 1 and 2). In contrary calibrated. Si, K, Ca, and Fe profiles highlight continuous vertical variations in quartz, illite/mica, calcite cement and chlorite, respectively that would otherwise be overlooked.

The log(Si/K) curve in the basal 7 cm of the studied bed (Fig. 2) declines upward, whereas K_2_O profile is mirror opposite of the log(Si/K) trend reflecting progressive increase in illite/mica content towards upper part of the bed. Log(Ca/K) illustrates bow-shaped trend and reflects formation of local diagenetic calcite cement in lower part of the bed. Log(Fe/K) shows a general upward decreasing trend. The upper 15 cm of the bed show that this bed may comprise two units, a lower mudclast-rich sandstone (having higher log(Si/K) than the upper mudclast-poor sandy mudstone (Fig. 2). Where mud clasts are present, they clustered closely and give serrate profiles (refer to core interval between 8-15 cm in Fig. 2). Such high-resolution details are seldom captured by petrographic analysis (refer to matrix and grain size profiles in Fig. 2), reflecting the superiority of Itrax core scanning technique over conventional methods.

## Conclusion

Although less often deployed with clastic rock cores, the Itrax scanning technology offers potential in terms of sub-mm scale mineralogical characterization of rocks, and in many respects it may be easier to apply to split rock cores as there are not the same issues with interstitial water and uneven core surfaces that complicate interpretation of soft sediment cores. Calibrated XRF core scanner data against well-characterized plug samples allow near-continuous vertical distribution and quantification of framework mineral grains, clay minerals, and diagenetic carbonate cements in the clastic rock cores to be documented. The analysis is carried out at significantly higher-resolution (micron scale) than is possible for plug sampling (cm to m scale). This is useful for clastic reservoirs particularly for fine-grained unconventional reservoir cores, with a simple framework mineralogy and low porosity, in which variable distribution of clay has a direct impact on fracking and subsequent fluid production.

## CRediT author statement

**Arif Hussain:** Conceptualization, Methodology, Validity tests, Data curation, Writing- Original draft preparation, Investigation. **Khalid Al-Ramadan:** Supervision, Writing- Reviewing and Editing, Resources.

## Data Availability

Data will be made available on request.
